# Is a diluted seawater-based solution safe and effective on human nasal epithelium?

**DOI:** 10.1007/s00405-020-06527-1

**Published:** 2021-01-03

**Authors:** Song Huang, Samuel Constant, Barbara De Servi, Marisa Meloni, Amina Saaid, Josip Culig, Marco Bertini

**Affiliations:** 1 Epithelix, Geneva, Switzerland; 2 Vitroscreen, Milan, Italy; 3 Laboratoire Fumouze, Levallois-Perret, France; 4 University of Applied Health Sciences, Zagreb, Croatia; 5 Laboratori Baldacci SpA, Pisa, Italy

**Keywords:** Nasal epithelium, Nasal hygiene, Isotonic seawater, Diluted seawater, Nasal irrigation, Rhinitis, Sinus health

## Abstract

**Purpose:**

Nasal irrigation is an effective method for alleviating several nasal symptoms and regular seawater-based nasal irrigation is useful for maintaining nasal hygiene which is essential for appropriate functioning of the nose and for preventing airborne particles including some pollutants, pathogens, and allergens from moving further in the respiratory system. However, safety studies on seawater-based nasal irrigation are scarce. In this study, the safety and efficacy of a diluted isotonic seawater solution (Stérimar Nasal Hygiene, SNH) in maintaining nasal homeostasis were evaluated in vitro.

**Methods:**

Safety was assessed by measuring tissue integrity via transepithelial electrical resistance (TEER). Efficacy was measured by mucociliary clearance (MCC), mucin secretion, and tissue re-epithelization (wound repair) assays. All assays were performed using a 3D reconstituted human nasal epithelium model.

**Results:**

In SNH-treated tissues, TEER values were statistically significantly lower than the untreated tissues; however, the values were above the tissue integrity limit. SNH treatment significantly increased MCC (88 vs. 36 µm/s, *p* < 0.001) and mucin secretion (1717 vs. 1280 µg/ml, *p* < 0.001) as compared to untreated cultures. Faster wound closure profile was noted upon pre-SNH treatment as compared to classical isotonic saline solution pre-treatment (90.5 vs. 50.7% wound closure 22 h after wound generation).

**Conclusion:**

SNH did not compromise the integrity of the nasal epithelium in vitro. Furthermore, SNH was effective for removal of foreign particles through MCC increase and for enhancing wound repair on nasal mucosa.

## Introduction

The nose is main entry point for inhaled air and the most exposed structure of the respiratory system, being in continuous contact with the external environment which contains air pollution including dust and allergens [1]. These particles are treated by nasal epithelium (including mucus) to limit their access to the lower respiratory tract and lungs [2]. Mucus (containing glycoproteins called mucins) or normal secretions from the epithelial cells lining the nasal cavity (nasal fossae) trap these particles that are either excreted by nose blowing, sneezing or pushed toward the pharynx (then swallowed) by cilia and their coordinated beating [1]. Keeping the nasal cavity clean may also generate—via reduction of nasal mucosal inflammation (rhinitis)—a larger airway, promote better sleep, and diminish onset of sinonasal diseases which would reduce work and school absenteeism.

Nasal hygiene can be maintained by regular irrigation using saline solutions which help to eliminate excess of secretions, pollutants, pathogens and allergens accumulating in the nasal cavity, reduce congestion and moisturize the nose [3]. Utilization of saline solutions for nasal irrigation has been shown to be easy to use, well tolerated and effective for nasal hygiene [4]. For example, in children, use of saline nasal irrigation has been shown to re-establish the post-rhinitis nasal permeability, prevent the recurrence of cold and flu and of nasal congestion [5]. Another clinical trial in children with allergic rhinitis (AR) has concluded that nasal saline irrigation may be a good adjunctive treatment option and that using nasal irrigation decreases the use of topical steroids for controlling AR in children, which may eventually lessen the side effects and economic burden of the condition [6]. Indeed, nasal irrigation with saline solutions are recommended as an adjunct therapy for common cold, acute and chronic rhinosinusitis and allergic rhinitis, alleviating sinonasal symptoms [7–10].

Saline solutions can be obtained by diluting seawater and used for nasal irrigation at isotonic concentration (0.9% NaCl) as this is equal to the concentration of Na^+^ and Cl^−^ ions in the human body [11]. In fact, early in twentieth century, microfiltered diluted ocean water (or “marine plasma”) has been shown to have identical ionic balance to that of blood serum and interstitial fluid [12]. In addition to sodium and chloride, diluted seawater contains the full spectrum of minerals and trace elements found in human plasma such as potassium, calcium, and magnesium. Studies have demonstrated the beneficial role of diluted seawater over classic saline solution preparations for nasal irrigation [13–15].

Although they have certain limitations, in vitro assays allow to study the primary mechanisms of action of the formulations and drugs which would otherwise be impractical to uncover and also gain deeper and more comprehensive information on the effects of treatments. The studies which aim to reveal the mechanism of action of the benefits of seawater-based solutions are rare.

In this study, an in vitro model (MucilAir™), which has previously been validated and utilized to study the toxicity of respiratory sensitizers [16, 17], has been used to assess the safety and efficacy of diluted seawater in maintaining nasal hygiene through transepithelial electrical resistance (TEER), mucociliary clearance (MCC), mucin secretion, and wound repair assays. TEER is a widely used method to functionally analyze tight junction dynamics in cell culture models of physiological barriers and epithelium permeability [18]. MCC represents a host defense mechanism of the airway epithelia to help clear foreign particles, pathogens, and chemicals [19, 20]. Mucin glycoproteins are the major constituents of mucus which lines epithelial surface and provide an important innate immune function by trapping and removing particles and pathogens from the airways via MCC [21]. Finally, wound healing is a technique used to investigate the effects of solutions on the re-epithelization of artificially generated in vitro wounds [22].

## Materials and methods

### Test product and the biological model

Stérimar Nasal Hygiene (SNH, Laboratoire Fumouze, Levallois-Perret, France) contains 31.82 ml seawater and purified water (qsp 100 ml) and is sterilized by microfiltration. Assays were performed in a 3D reconstituted human nasal epithelium model, MucilAir™ (Epithelix Sàrl, Geneva, Switzerland), a mixture of human nasal cells isolated from a panel of 14 different donors. Cells were cultured in 500 µl of MucilAir™ culture medium in a CO_2_ incubator (37 °C, 5% CO_2_, 100% humidity, Heracell, Waltham, Massachusetts, United States) in 24-well plates with 6.5-mm Transwell^®^ inserts (Corning Life Sciences, Corning, New York, United States). Before treatments, inserts were washed with 200 µl of MucilAir™ culture medium and the quality of the tissue was assessed under an inverted microscope (Zeiss Axiovert 25, Oberkochen, Germany).

### Treatments

For TEER, MCC, and mucin secretion, Mucilair™ tissues were left untreated or were treated with SNH (10 µl) twice a day with an 8-h interval, for 4 days. SNH was applied on the apical side of the epithelium in 24-well plates. Each day, culture medium was frozen at -80ºC for further analysis. Assays were performed with samples collected at Day 1 and Day 4 of treatment. The details of each experiment are described below:

### TEER

Tissues were left untreated (*n* = 3) or treated with SNH (*n* = 3) for up to 4 days. Measurements were performed using a Millicell ERS voltohmmeter (Millipore, Burlington, Massachusetts, United States). Three measurements were performed per sample where the basal value was the measurement performed at Day 0. The resistance of the tissue was calculated by subtracting the blank resistance (insert with no tissue) from the read-out resistance (mean of three) and multiplying by the epithelium surface size (0.33 cm^2^).

### MCC

To evaluate the effects of SNH on the clearance of impurities, microbeads (5 µm) were added onto the apical surface of untreated (*n* = 3), SNH-treated (*n* = 3), and 50 µM isoproterenol-treated (positive control, *n* = 3) tissues. Isoproterenol is a bronchodilator, known to activate ciliary beat frequency without deleterious effects [23, 24]. For bead tracking, one-minute videos (images taken every second) were recorded using DMIRE2 microscope (Leica, Wetzlar, Germany) equipped with DS-5MC camera (Nikon, Tokyo, Japan). 200–500 beads were tracked (Image Pro Plus, Media Cybernetics, Rockville, Maryland, United States).

### Mucin secretion

Tissues were left untreated (*n* = 3) or treated with SNH (*n* = 3) for up to 4 days. Mucin secretion was evaluated using an enzyme-linked lectin assay method. 60 μl coating solution (250 μg/ml lectin in PBS pH 6.8) was dispensed in 96-well NUNC Maxisorp plates (Thermo Fisher Scientific, Waltham, Massachusetts, United States) and incubated 1 h at 37 °C, 0.5% CO_2_. Plates were washed 3 times with 200 μl high salt PBS (HS-PBS, PBS-0.5 M NaCl–0.1% Tween 20). Samples were sonicated 10 min at 35 °C. Samples and 50 μl of standards at various dilutions (Mucins from bovine submaxillary gland, Sigma, Cat no: M3895, St. Louis, Missouri, United States) were dispensed into wells. Plates were incubated for 30 min at 37 °C, 0.5% CO_2_ and washed with HS-PBS. 50 μl detection solution (1 μg/ml of Glycin max soybean lectin-Horseradish Peroxidase conjugated, in 0.1% BSA- PBS) was added to each well and plates were incubated for 30 min at 37 °C, 0.5% CO_2_. Plates were washed 3 times with HS-PBS. 50 μl/well of TMB substrate reagent (BD OptEIA™, Cat no: 555214, BD Bioscience, Franklin Lakes, New Jersey, United States) was then added to each well and plates were incubated in the dark for 15 min at room temperature. The reaction was stopped with 50 μl/well of 2 N H_2_SO_4_ and plates were read at 490 nm.

### Wound repair assay

Tissues were treated with classical saline solution (0.9% NaCl) or SNH for 30 min (30 µl, *n* = 3) before generating an injury with a glass capillary. The injury procedure was controlled on the saline solution-treated tissues by TEER. The products were removed after injury and immediately re-applied to eliminate floating cells. The wound areas were quantified from images acquired by bright-field microscopy using Image J (209 pixels = 100 μm). The percentage of post-injury re-epithelialization was compared with the first acquisition (immediately after wound) of each product. Two tissues for each condition were evaluated.

## Results

### Evaluation of tissue integrity

**Fig. 1:**
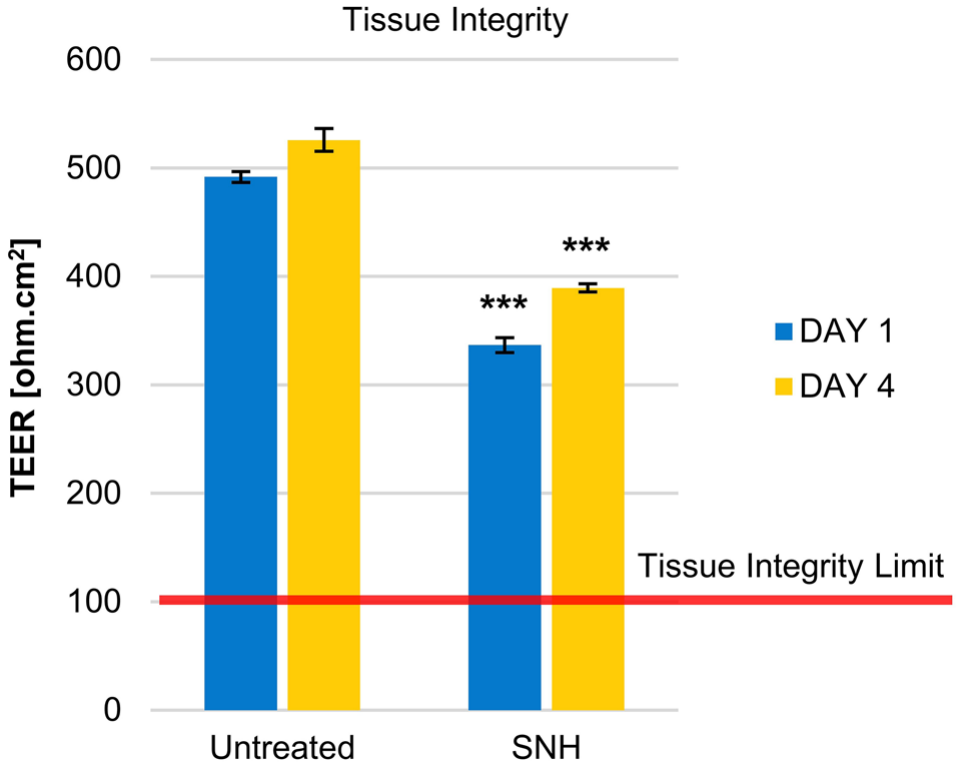
Effects of SNH on tissue integrity. Effect of SNH treatment on tissue integrity in comparison to untreated tissues was monitored by transepithelial electrical resistance (TEER) on Days 1 and 4. ***p < 0.001 compared to untreated cultures

After treatment with SNH, a significant decrease in TEER was observed at both Day 1 and Day 4 (Fig. 1). Nevertheless, these values were within the range of an intact epithelium (200 and 600 Ω.cm^2^), indicating that SNH treatment does not compromise epithelial integrity.

### Mucociliary clearance

**Fig. 2:**
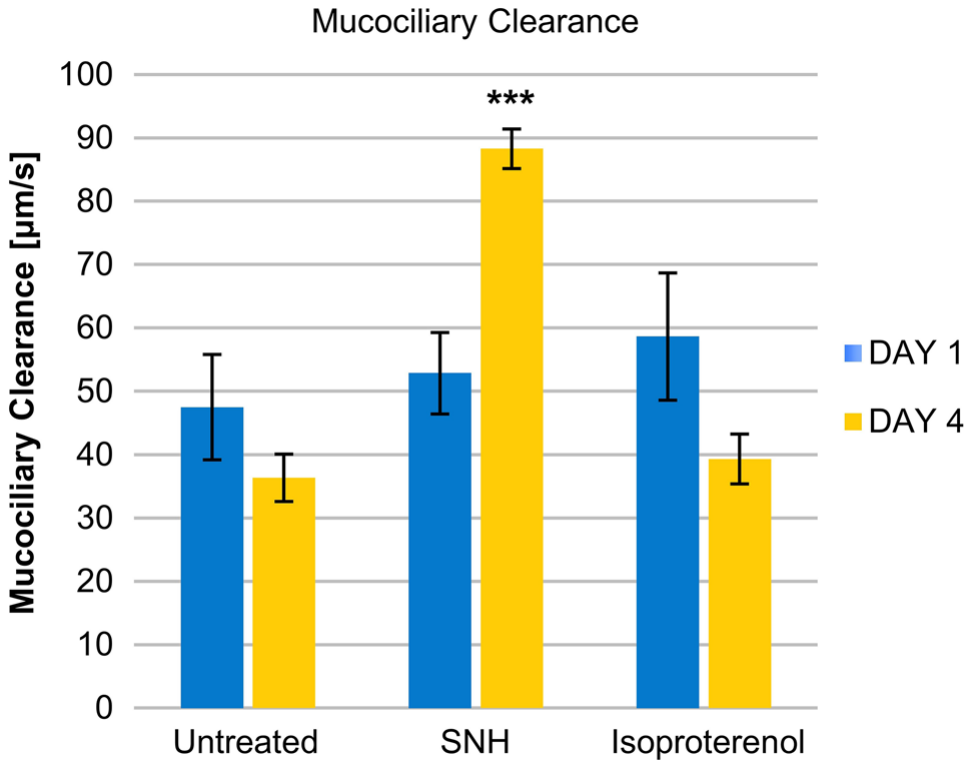
Effect of SNH on mucociliary clearance. Muciliary clearance (MCC) rates were monitored in untreated, SNH-treated and isoproterenol-treated tissues on Days 1 and 4 of treatment. ***p < 0.001 compared to untreated cultures

As shown in Fig. 2, after one day of SNH treatment*,* there was no significant difference in MCC rates compared to untreated cells. However, at Day 4, there was a significant increase in the microbead clearance velocity of epithelial tissues treated with SNH compared to untreated (88 vs. 36 µm/s, *p* < 0.001), even higher than the one observed in tissues treated with isoproterenol.

### Mucin secretion

**Fig. 3:**
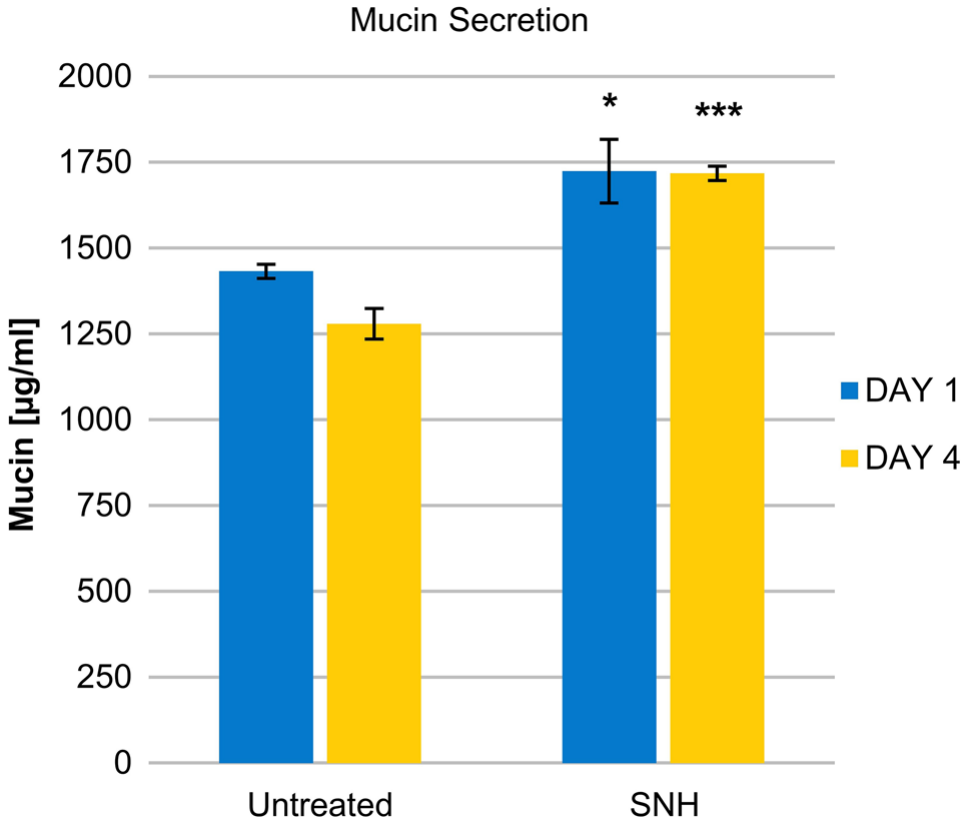
Effect of SNH treatment on mucin secretion. Untreated and SNH-treated tissues monitored on Days 1 and 4 of treatment. *p < 0.05, ***p < 0.001 compared to untreated cultures

Treatment with SNH caused a strong increase in the levels of secreted mucins from nasal epithelium cells starting from Day 1 after treatment compared to untreated cells (*p* < 0.05, Fig. 3). On Day 4, the high secretion of mucins was maintained, at levels significantly higher than in the untreated cells (*p* < 0.001).

### In vitro wound repair

**Fig. 4:**
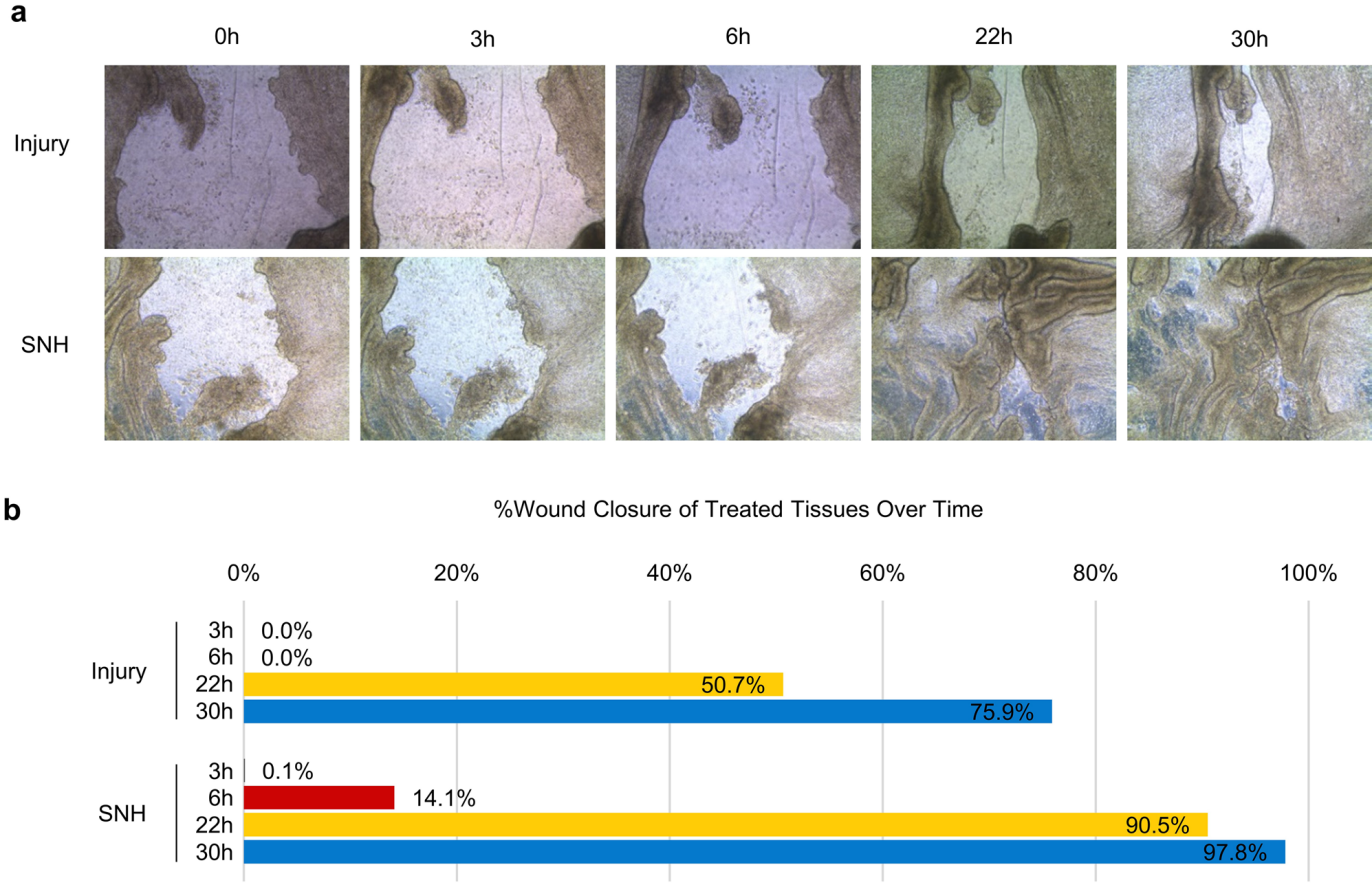
Evaluation of the wound repairing capacity of SNH compared to saline solution. **a** Bright field selected images of tissues at the beginning (0 h) and 3, 6, 22, and 30 h after treatment with SNH or saline solution. **b** Quantification of wound closure (re-epithelialization) of treated tissues in **a** at indicated time points

As shown in Fig. 4, 22 h after generation of the injury, the wound in SNH-pre-treated tissues had achieved a 90% closure. In contrast, the wound in the saline-solution-pre-treated tissues had only reached a 50% closure. Moreover, at the end of the experiment (30 h), the wound in SNH-pre-treated tissues was reaching complete closure (97.8%). This indicates an almost complete re-epithelization, while at the same time-point a 75% closure only was observed in the control tissues.

## Discussion

Nasal irrigation with saline solutions can alleviate sinonasal symptoms and is often recommended as an adjunct therapy in several sinonasal conditions and for nose-hygiene maintenance [25]. Clinical trials have shown that nasal irrigation with seawater solutions could re-establish the proper function of the nose, help prevent the repetition of allergic rhinitis episodes, and lessen the accompanying side effects of medications and their associated economic burden [5, 6]. In the present study, in vitro safety and efficacy of an isotonic diluted seawater solution (SNH) on maintaining nasal hygiene has been evaluated.

Tissue integrity after SNH application was evaluated by TEER assay which assesses functioning of tight junctions in cell culture models [26]. Upon treatment with SNH, TEER values were within the range of 300–400 Ω cm^2^, accepted for intact epithelium, demonstrating that SNH is not affecting the integrity of the tissue. The observed variations are probably due to ion channel activity modulations with no toxic effect on the airway epithelium.

Original experiments on the efficacy of SNH have been included in the present report. The airway epithelium represents a primary defense mechanism against respiratory challenges and functions as a physical barrier and immune response modulator from the nostrils to the lung by means of MCC [27]. MCC relies on mucus secretion and cilia beating and its measurement expresses the rate of foreign particle, pathogen, and chemical clearance. SNH significantly increased MCC at Day 4 in comparison to untreated cultures. Overall, these data suggest a beneficial effect of SNH on MCC which is compromised in sinonasal diseases such as chronic rhinosinusitis [28].

Additionally, levels of secreted mucins were measured. Treatment with SNH caused a strong increase in the levels of secreted mucins 4 days after treatment compared to untreated cells (Fig. 3). Since MCC is an innate mechanism to clear nasal secretions, these results suggest that SNH can contribute to maintenance of a good nasal hygiene.

Many sinonasal pathologies can be accompanied by small wounds, crusts or bleeding in the nose [29, 30]. These wounds are repaired through a highly coordinated process which includes basal cell spreading, migration, proliferation, and differentiation [31, 32]. This ensures repair and regeneration of the airway epithelium, which are crucial for sustaining the epithelial barrier function [33]. To evaluate the effect of SNH on wound repair, an in vitro wound repair assay was performed. SNH is shown to act faster than saline solution in promoting the onset of re-epithelization of nasal tissues after injuries. The wound repair capacity of SNH was evident at just 22 h (> 90% wound closure was observed) while the tissue reached almost complete closure at 30 h (97.8%), which outlines the benefits of SNH on tissue re-epithelization after wounding.

Altogether, these in vitro data suggest that SNH is safe for human use and is effective in increasing the cleansing of nasal cavities, promoting wound re-epithelization, thus sustaining a proper nasal epithelial structure and hygiene.

## Conclusions

Using an in vitro 3D human nasal epithelium model, this study demonstrates that SNH, an isotonic diluted seawater solution, is safe on nasal epithelial cells and effective on enhancing the rate of mucociliary clearance and speed of wound healing in the nasal cavity. These data support the available evidence that regular nasal irrigation with isotonic saline solutions is beneficial for maintaining the hygiene of the nose which, in turn, may reduce the occurrence of episodes of various sinonasal conditions.
